# Detection of Homologous Blood Transfusion in Sport Doping by Flow Cytofluorimetry: State of the Art and New Approaches to Reduce the Risk of False-Negative Results

**DOI:** 10.3389/fspor.2022.808449

**Published:** 2022-02-10

**Authors:** Francesco Donati, Xavier de la Torre, Sarajane Pagliarosi, Daniela Pirri, Giuliana Prevete, Francesco Botrè

**Affiliations:** ^1^Laboratorio Antidoping, Federazione Medico Sportiva Italiana, Rome, Italy; ^2^REDs–Research and Expertise in AntiDoping Sciences, Synathlon, Quartier Centre, ISSUL–Institute of Sport Sciences, University of Lausanne, Lausanne, Switzerland

**Keywords:** homologous blood transfusions, human blood groups, red blood cell surface antigens, flow cytometry, doping control

## Abstract

This article presents the results of a study aimed to give new suggestions and strategies for improving the implementation of the flow cytofluorimetry-based method for the detection of homologous blood transfusions in doping control. The method is based on the recognition of the phenotypic mismatch between minority blood group antigens possessed by the donor and the recipient. Two strategies have been followed to reduce the risk of false-negative results: (i) the monitoring of a broader range of erythrocytes surface antigens; and (ii) the application of different surface erythrocyte staining protocols, tailored on the different antigens and the type of antigenic mismatch that had to be detected (whether it is the donor or the recipient who expresses or not the antigen to be detected). Special attention has also been focused on the time factor, to avoid prolonged sample storage, since hemolysis may have a significant impact on the reliability and quality of the results. Our experimental evidence suggests that the risk of false-negative results can be minimized by (i) the expansion of the antigen panel, with the inclusion of four additional targets; (ii) a more accurate selection of the gating area of the red blood cells; (iii) the choice of a better fluorochrome (alexa fluor 488) to be conjugated to the secondary antibody; and (iv) the implementation of different staining protocols depending on the nature of the double population to be detected (donor expressing vs. recipient non-expressing and vice versa). The combination of the above approaches allowed a significant reduction of false-negative results, assessed on samples simulating a homologous blood transfusion between two compatible subjects.

## Introduction

“Blood doping” is a prohibited practice used by cheating athletes to increase the blood capacity to transport oxygen to the tissues, and it can indeed represent a critical advantage among elite athletes, especially in endurance sport disciplines. Blood doping can be achieved by a variety of non-physiologic methods, and hence are banned by the World Anti-Doping Agency (WADA).

In the last decade WADA-accredited laboratories have developed testing methods for the detection of the many different forms of blood doping, including the administration of recombinant erythropoietin (EPOs) (Lasne and de Ceaurriz, 2000; Arndt and Kumpel, 2008; Lasne et al., 2009), plasma volume expanders (PVEs) (Thevis et al., 2000; Mazzarino et al., 2010), artificial hemoglobin based oxygen carriers (HBOCs) (Goebel et al., 2005; Simitsek et al., 2007; Donati and Botrè, 2010; Staub et al., 2010; Donati et al., 2011), allosteric effectors of hemoglobin (like efaproxiral, RSR13) (Breidbach and Catlin, 2001) and also homologous blood transfusions (HBT) (Nelson et al., 2003; Giraud et al., 2010). Specifically, HBT represent one of the illicit methods that athletes currently pursue to improve the oxygen supply to the tissues and consequently sports performance. HBT was initially abandoned by athletes who began to use recombinant erythropoietin with the aim of increasing the number of circulating red blood cells, but the development and implementation of a direct method to detect recombinant human erythropoietin (rHuEPO) has caused blood transfusions to regain interest among cheating athletes. A method for the detection of HBT has been implemented by WADA laboratories, while no internationally recognized method has been finalized so far for the direct detection of ABT, which can at present be revealed only indirectly by the longitudinal strategy of the athlete biological passport (ABP) which targets key blood parameters (Saugy et al., 2014), and/or by the detection of synthetic components of the plasticizers used for the production of the blood bags that may be released into the blood stream (Monfort et al., 2010). The anti-HBT method was initially developed in 2003 (Nelson et al., 2003) and adopted for the first time on the occasion of the antidoping tests at the Athens Olympic games in 2004. Some cases of HBT abuse among athletes were discovered in time period 2004–2008. Starting from 2008, the lack of HBT cases among athletes raised the suspicion that cheating athletes may have switched from HBT to ABT practices (Lamon et al., 2009). However, the evidence of HBT cases discovered during the recent Tokyo 2020 summer Olympics showed that HBTs are still being used by athletes for doping purposes (The International Testing Agency, 2020).

The method of detection of HBT is based on the recognition of two, phenotypically distinct, red blood cells (RBCs) populations, within the athlete's blood, based on the presence of minor blood group antigens on the erythrocytes surface. According to the different blood groups, antigens can be proteins, but also glycoproteins, glycolipids and glycopolysaccharides (Maton et al., 1993). At present, the International Society of Blood Transfusion (ISBT) recognizes 30 major blood groups, including the principal ABO and Rh systems, and it is well-known that human blood group polymorphisms can vary among human ethnic groups. Among the 30 blood groups, over 300 different antigens were identified, most of which are very rare and expressed only by certain ethnic groups. For instance, B blood group has a higher frequency in central Asia and India, while it is much less common in other geographical areas (Daniels et al., 2007).

The cytofluroimetric method of detection of RBCs double populations uses monoclonal antibodies and polyclonal antisera directed against the antigens of the transfused sample (Giraud et al., 2008). A primary (capture) antibody binds the antigen (if expressed) on the RBC surface. A secondary (detection) antibody, conjugated to a fluorochrome, is then used to bind to the primary antibody, so allowing the detection by flow cytofluorimetry. WADA did not issue a technical document formally detailing the criteria to issue an Adverse Analytical Finding (AAF) for homologous blood transfusions: however, the consensus is that, to highlight a phenotypic mismatch between the donor and the recipient, the following conditions must be met: (1) an evident separation among double erythrocytes populations must be visible in the fluorescence histogram for at least two antigens, (2) the number of events of the minority (donor) peak must greater than a threshold number of red blood cells (120, according to our validation study) and (3) the signal to noise ratio (S/N = number of events of the minority peak divided by the number of background events in the same gating area) must be >3. Although the method has excellent specificity (100%) and sensitivity (down to 0.2% of the donor cells into the recipient may be detected) (Giraud et al., 2008), some critical issues have emerged over the years, such as a significant percentage of false-negative results. This is the case, for instance, in which both the donor and the recipient share the same panel of surface antigens. Furthermore, it is possible that, even in the case there is a mismatch between the donor and the recipient for one or more antigens (i.e., one expressing and the other non-expressing the considered antigen), this discrepancy may be not unambiguously detected, since double erythrocyte populations in which the two fluorescence peaks are not clearly separated are considered unclear/uncertain and reported as “negative for HBT.” Moreover, as said above, the positivity criteria for a sample (which is screened for several minor blood group antigens) requires at least two mismatched antigens. Therefore, the specificity and the sensitivity of this method to detect mixed RBCs populations is depending on the number of antigens analyzed. Clearly, by broadening the panel of target antigens the discrimination between donor and receiver's blood improves. A base panel of 8 antigens (namely big C, small c, big E of the Rh system, Jka and Jkb of the Kidd system, Fya and Fyb of the Duffy system, and big S of the MNS system) was initially considered for the screening test. Despite the good characteristics of specificity and sensitivity, the method nevertheless presents some limitations which can be summarized as follows:

1) It might be possible that both donor and the recipient share the same pattern for the panel of antigens screened. In this case, there cannot be any mismatch between donor and recipient's antigens and it is not possible to detect any double erythrocyte population, a circumstance that can determine the occurrence of “false negatives” results (Krotov et al., 2014).
2) A critical issue concerns the stability of blood samples that are subjected to the staining procedure with the antibodies. The currently in use method provides that the volume of whole blood collected for the anti-doping test is divided in two aliquots (A and B) that are sent to the laboratory for the analysis. The A aliquot is taken in a small part to be stabilized and then used, while the rest is stored in the fridge; while the B aliquot remains sealed and immediately stored at 4°C, to be used just in case of the counter analysis is requested by the athlete following an AAF reported for the A aliquot. By considering the analysis timing, it is possible that, in case of positivity ascertained on sample A, the B-analysis might be performed after a period of time such that the sample may have undergone modifications during the period of storage. Consequently, the detectability of double erythrocyte populations can be seriously compromised in the cases of prolonged storage time (Lima et al., 2021).
3) The expression of blood group antigens on the surface of red blood cells is genetically determined. The number of antigenic sites on the surface of red blood cells varies between individuals based on the presence of genetic polymorphisms, whose relevance is at present not considered under the WADA rules. This aspect has repercussions on the real ability of the method to separate and identify the cell populations of the donor and recipient in an optimal way. Also in this case, the result may be the generation of false negative samples.

In the view of the above, we propose the following additions to improve the detection of HBT by flow cytofluorimetry, by specifically considering the following:

1) To evaluate a broader number of antigens, with the aim to improve the sensitivity of the method, thus reducing the number of possible false-negative results. Data obtained were used to assess the frequency of blood group antigens in different ethnic groups so as to determine the most informative antigens to incorporate.
2) To explore new strategies for the qualitative improvement of the staining of erythrocyte antigens in order to improve the separation between the donor and recipient erythrocyte populations in the most difficult cases.
3) To carry out a stability study to determine the impact of blood sample storage time on the ability to detect double erythrocyte populations.

## Materials and Methods

### Staining of the Blood Antigens

Two hundred microliter of EDTA-anti coagulated human whole blood were stabilized by adding 2 mL of CellStab stabilization buffer (Diamed GmbH, Biorad). The stabilized sample is counted for the number of RBC (#RBC) by using a Sysmex XN-1000 hematology analyzer and subsequently diluted in PBS buffer (1%BSA). Primary antibodies and antisera were obtained from DIAMED (Gmbh, Cressier Switzerland): monoclonal IgM anti big C, small c, big E, Jka, Jkb, and serum IgG anti Fya, Fyb and big S. All antibodies were previously titred in order to use them at the optimal concentration for the staining. Fifty microliter of the titrated primary antibodies were added to 100 μL of a blood diluited sample containing 5 million RBC. Samples were incubated for 90 min at room temperature and under mild agitation. At the end of the incubation the samples were washed with Diluent N.A. solution (Diamed GmbH, Biorad) in order to discard the excess of primary antibody, then 50 μL of the secondary antibodies were added to all samples (Goat anti-human IgM-FITC or PE or AlexaFluor 488 conjugates (Invitrogen, Thermo Fisher) and Goat anti human IgG (H + L)-FITC or PE or AlexaFluor 488 (Invitrogen, Thermo Fisher). Samples were incubated in refrigerator at 4°C for 45 min and subsequently washed with Diluent N.A. solution (Diamed GmbH, Biorad). The RBC pellet was diluted in 1 mL of PBS (1%BSA) for the subsequent acquisition on the flow cytometer. Samples were analyzed on FC500 Beckman Coulter Flow Cytofluorimeter. Platelets and other small events were excluded using an appropriate discriminating factor on the forward scatter (FS) channel. Red blood cells were appropriately gated from the other cell populations in a FS vs. side scatter (SS) dot plot. An anti-Glycophorin-a (CD235a-FITC) antibody (Beckman Coulter) was used in the staining, to clearly identify homogenous red blood cells population, excluding interfering aggregates from the acquisition. Fifty thousand events were collected for each sample.

### Broadening of the Antigenic Panel

A total of 595 whole blood samples were considered in this study. All samples were collected in the framework of official anti-doping tests analyzed by the Antidoping Laboratory of Rome, Italy, for homologous blood transfusion and/or athlete biological passport, and can therefore be considered to be collected from athletes of European/Caucasian origin. All whole blood samples were selected from those collected from athletes expressing their consent, on the doping control form, to their use for research purpose. All samples were analyzed targeting the eight antigens of the initial screening panel (big C, small c, big E, Jka, Jkb, Fya, Fyb, big S). A subset of 41 samples, chosen randomly, were also screened for four additional antigens, not included in the original protocol: small e (Rh system), big K and small k (Kell system), and small s (MNS system). All 595 samples were selected after a prior hemoagglutination screening of their major blood group phenotype since they resulted to be O + (ABO Rh), this way to consider the possibility of making HBT mixtures among all the samples of the database.

Two databases of antigenic haplotypes were constructed: a first database considering the eight antigens of the base panel, and an extended database considering also the new four antigens for a total of 12. Frequencies of expression of blood antigens were calculated using Microsoft Excel software.

### Stability Study

The stability study was performed over a time period of 60 days, i.e., realistically, the maximum conservation period for a blood sample at 4°C. Eleven blood mixtures were prepared *ex vivo* in the laboratory by considering a donor percentage of 5% (to simulate a situation in which the transfusion has taken place recently) and 5 more mixtures were prepared at 0.5% (to simulate a scenario of homologous transfusion which has taken place long time before). Mixtures were prepared starting from anonymous EDTA-anticoagulated whole blood and stored refrigerated (2–8°C) in eppendorf tubes.

The mixtures were analyzed using the base panel of eight antigens within 24–48 h of their preparation (*t* = 0), after 30 days of storage in the refrigerator (*t* = 1) and after 60 days of storage (*t* = 2).

### RBC Staining by Using Different Fluorochromes-Conjugated Secondary Antibodies

For each antigen of the base panel of 8 antigens, 10 mixed samples (with donor at 5%) were prepared by carrying out combinations in order to have five different mixtures with the recipient non-expressing the target antigen (Double RBC population recipient non-expressing, DPN) and five different mixtures with the recipient expressing the target antigen (Double RBC population recipient expressing, DPE). Different fluorochromes-conjugated secondary antibodies were used for the comparative analysis (fluorescein isothiocyanate FITC that is currently fluorochrome of choice for routine analysis, then phycoerythrin PE and Alexa Fluor488 were also used). For each secondary antibody conjugated to a different fluorochrome, the concentration of use was standardized (0.05 mg/mL each staining). The detection antibodies were used alone or additively in pairs between fluorochromes having different maximum of emission so that they can be detected by two different flow cytometry detectors (PE+FITC on detector FL1 with maximum emission at 525 nm and PE + AlexaFluor on detector FL2 with maximum emission at 575 nm). This combination of fluorochromes was considered in order to understand whether the additivity of the signals can contribute to obtaining a better separation between the red blood cells of the donor and those of the recipient.

### Gating Strategies

Three different RBC gating strategies have been applied to the FS/SS dual plot of the flow cytofluorimeter. One gating area was set to enclose the “core” of the mature RBC cloud. A second area was set include both the core of the mature RBC cloud and its entire tail where immature reticulocytes are detected. A third, tailored and reduced area was set to circumscribe only the transfused RBC.

## Results and Discussion

### Frequency of Expression of Eight Base Panel Antigens Used for the HBT Screening Test

Relative frequency of expression of the base panel, eight antigens has been calculated on the whole database. Antigen Big E showed the lowest expression as it is the only antigen which expression resulted lower than 50% in the individuals (24%), while antigen Jka showed the highest expression frequency (77.5%). The frequency of the other antigens are as follows: big C 67.5%, small c 75.3%, Jkb 70.4%, Fya 58.8%, Fyb 76.6%, big S 51.3%. Except for big E and Jka, the other antigens have expression frequency not far from the ideal 50%. These frequencies are in line with those obtained by another research group, who analyzed a total of 535 Russian (Caucasian) athletes of various sports and disciplines (Krotov et al., 2014).

Theoretically, an antigen is more informative when its frequency, within the population, is 50% or very close to it. The frequency of 50% gives the highest probability that blood samples from two different individuals, when mixed together, can be differentiated for a certain antigen thus obtaining the detection of two distinct populations of red blood cells (one of the donor and one of the recipient). Antigen frequencies close to 100% (all population individuals express the antigen) or close to 0% (all population individuals non-expressing the antigen) are much less informative, since the probability to differentiate mixed samples strongly decreases. Data here obtained prove that the base panel of eight antigen that we routinely apply for the screening is perfectly suitable for the analysis of samples delivered to the laboratory, being in general from subjects of European/Caucasian origin.

For the base panel, we detected 103 different combination or haplotypes (in this particular case, with the word “haplotype” we intend the combined phenotype expression of each antigen) on a total of 595 samples analyzed, 18 of which are unique combinations carried by only one individual of the database. The most common combination of antigens in the database is the following: big C+, small c+, big E–, Jka+, Jkb+, Fya+, Fyb+, big S+ (where + means expression and—means non-expression) which is shared by 26 different individuals (Table 1). From a statistic point of view, the maximum number of different combinations of haplotypes in a database of eight antigens is 2^8^ = 256 different combinations. This is the consequence of the fact that each antigen can be, or not be, expressed on the RBC surface and there is no expression linkage among any of the antigens (the expression/non-expression of each antigen does not depend on the expression/non-expression of any other antigen). We have detected a total of 103 different haplotypes on a possible maximum number of 256 (40.2%). Nevertheless, the presence of shared combinations of antigen haplotypes is the key factor that determines the possible occurrence of false-negative results, since the possibility of individuals sharing the same matched antigen haplotype or carrying only one antigenic difference is not remote.

**Table 1:** Most frequent and common haplotypes shared by the individuals of the database (+ = expression, – = non-expression of the antigen).

**Haplotype**	**Big C**	**Small c**	**Big E**	**Jka**	**Jkb**	**Fya**	**Fyb**	**Big S**	**Abs fr**.	**Rel fr**.
1	+	+	–	+	+	+	+	+	26	0.044
2	+	–	–	+	+	+	+	+	24	0.040
3	+	+	–	+	+	+	+	–	22	0.037
4	+	+	–	+	+	–	+	+	18	0.030
5	+	+	–	+	+	–	+	–	18	0.030
6	+	+	–	+	–	–	+	–	15	0.025
7	+	–	–	+	+	+	+	–	15	0.025
8	–	+	+	+	+	+	+	–	13	0.022
9	–	+	–	–	–	–	–	–	13	0.022
10	+	+	–	+	–	+	+	–	12	0.020
11	+	+	–	–	+	–	+	+	12	0.020
12	–	+	+	+	–	+	+	+	11	0.018
13	–	+	–	+	+	+	+	+	11	0.018
14	+	+	–	+	–	–	+	+	10	0.017
15	+	–	–	+	+	–	+	+	10	0.017
16	+	–	–	–	+	+	+	+	10	0.017

**Figure 1:**
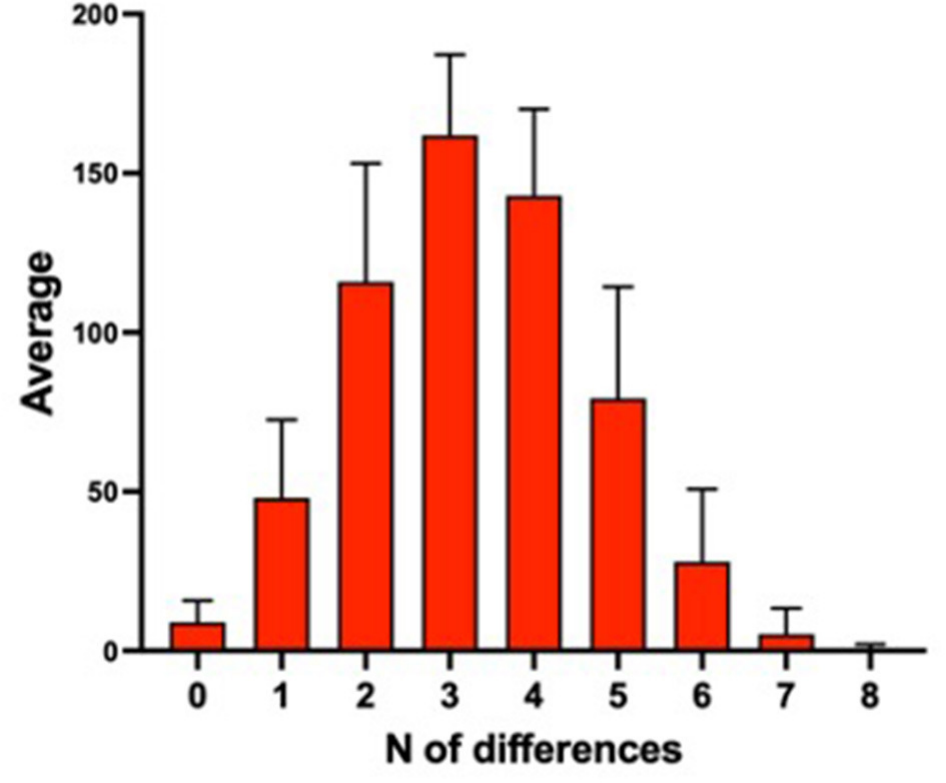
Mean number of antigenic differences among 595 samples of the database.

Subsequently, each individual was compared to all the others to calculate the mean number of differences in the shared antigens (the samples sharing the same expression haplotype would consequently have 0 antigenic differences). Results are shown in Figure 1.

The majority of the samples showed three or four antigenic differences when compared to the other samples of the database. Moreover, a little less than 1/10 (9.3%) of the individuals had zero or only one antigenic difference with another subject in the database. These individuals might reciprocally transfuse their blood without giving rise to an AAF with the current criteria. A sample resulting only one antigenic mismatch is declared “suspicious” and the laboratory reports the result as an “atypical finding” (ATF). These data highlight the importance of broadening the antigenic panel in order to lower the number of possible “false-negative” cases.

### Frequency of Expression of Antigens Used for the HBT Screening Test on a Global Scale

**Figure 2:**
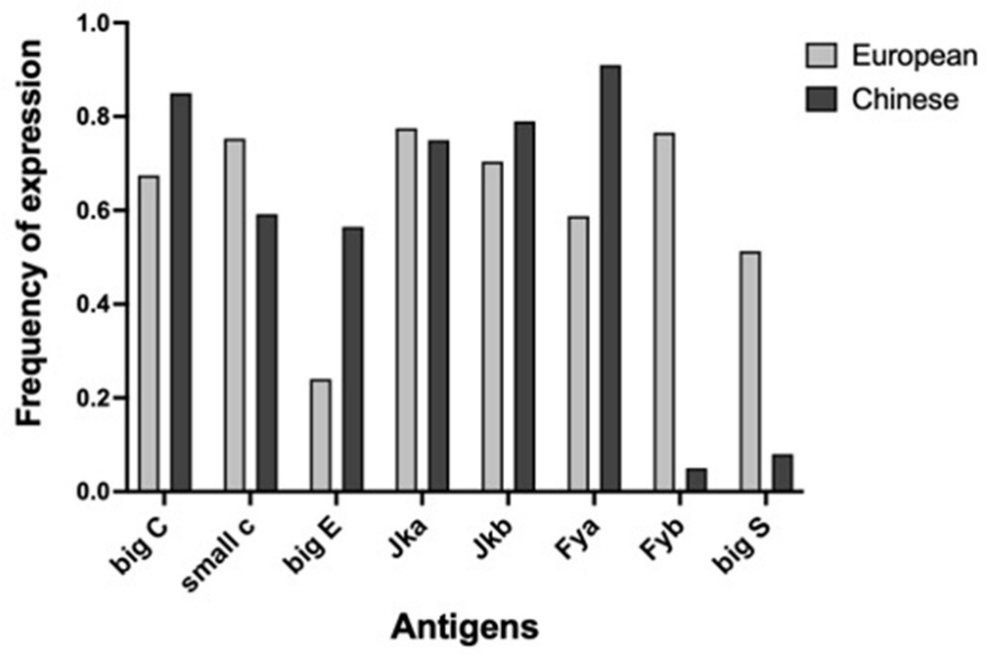
Comparison between the expression frequency of human minor blood group antigens of European database (gray) and Chinese database (black). Marked differences are present regarding to Fya, Fyb, big S, and big E antigens.

It is well-known that human blood group phenotypes are variable in human populations since ethnic differences exist in the expression frequency of blood phenotypes. Ethnic differences in the frequency of expression of Duffy antigens in the Chinese population compared to non-Chinese population were reported in literature (Chunji et al., 2009). For instance, the antigens Fyb and Big S are highly expressed in the European population with a frequency of distribution higher than 50%, whereas their expression in the Chinese population is <10%. As a consequence, antigens carrying very high and/or very low frequency of expression were not suitable to construct a reliable screening panel for doping control within the Chinese population. We compared antigen frequencies of our database (which, as already pointed out, can be considered to be basically constituted by individuals of European/Caucasian origin) with the results reported for the Chinese population (Chunji et al., 2009). Results are shown in Figure 2.

A very marked difference emerges in the frequencies of Fyb and big S antigens (and of less extent for big E and Fya). In our “European” database, antigen Fya has an expression frequency of 59% (close to the optimal 50%) while in the “Chinese” database has a very high frequency (91%). Antigens Fyb and big S have a frequency, respectively, of 76 and 51% in our database, while in the Chinese database their frequency is 5 and 8%, respectively.

We also checked for the presence of common repeated haplotypes shared by the European and Chinese databases. A first difference between these two databases is that the repeated haplotypes for our European database account for 60.4% of all haplotypes while the Chinese database account for 81.4%. This means that in the European database there is a higher number of unique haplotypes that is a consequence of a higher variability within the population. This is a typical consequence of the presence of antigens expressed at very high frequency (such as Fya) or very low (such as Fyb and S) within a Chinese population. These data would suggest that the use of Fya, Fyb, and S antigen is not the best choice in building a base panel of antigen to detect homologous blood transfusion within a Chinese population, while these three antigens are suitable for creating a suitable panel of antigen to be used for European samples. This also is corroborated by the fact that common haplotypes in the European database are not the same common haplotypes in the Chinese one and that is another clear indication that the method adopted must take into the account the ethnic variability among the ethnic groups. Hence the importance of investigating polymorphisms at regional scale emerges.

### Broadening the Panel of Antigens for the Screening Test

**Figure 3:**
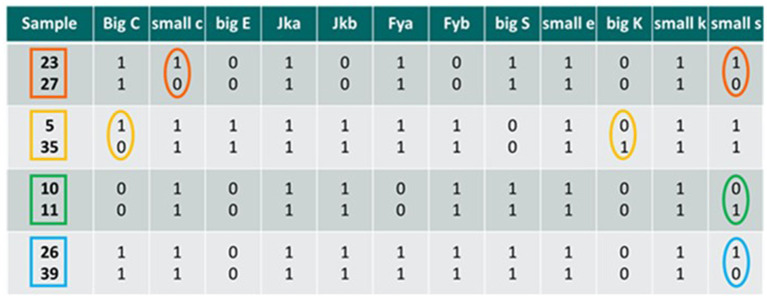
Comparisons between mixed samples; the antigenic differences detectable by the method are highlighted in circles in the case a transfusion occurs between the two individuals considered. 0, antigen non-expressed; 1, antigen expressed. Big C, small c, big E, Jka, Jkb Fya, Fyb, and big S are the base panel antigens, while small e, big K, small k, and small s are the new antigens selected.

A subset of 41 samples were screened for four supplementary antigens, namely small e (Rh system), big K and small k (Kell system), and small s (MNS system) so obtaining an extended panel of 12 blood group antigens. Despite the considerable number of antigens present on red blood cell membrane surfaces, the selection for their use in this flow cytometric method is very rigorous and leaves little freedom of choice. First of all, it should be considered that primary antibodies are not commercially available for all antigens. Secondly, these antibodies are developed for *in vitro* immunohematology methods and not for flow cytometry, consequently making it mandatory to optimize the reagents before performing the staining. Another issue is represented by the number of antigenic sites present on each RBC; this number can be sometimes so low that it does not allow adequate staining such as not to be able to highlight a mixed erythrocyte population in the sample under examination (Arndt and Kumpel, 2008). Therefore, we selected four new antigens based on the following two parameters: (i) their expression on the erythrocyte plasma membrane, and (ii) the high repeatability of their staining during preliminary tests. Also, the supplementary antigens were preliminarily validated by analyzing double populations at different donor percentages, to verify that the donor detection sensitivity was comparable to that of the base panel antigens (0.25%). Despite the incidence frequencies of the four antigens are far from the optimal 50% (i.e., 100% for small e and small k antigens, 90.2% for small s antigen, and 2.4% for big K antigen), the addition of these new antigens allowed to significantly reduce the number of false-negatives results. This reduction was highlighted by making pairwise comparisons between all the 41 samples tested, hypothesizing transfusions between the members of each pair, and calculating the differences between one sample and another, initially considering only the antigens currently in use, and subsequently extending the antigenic panel. By utilizing the standard panel of eight antigens we calculated a total of 164 possible combination that can give a “false negative” result, while utilizing the extended panel of 12 antigens the number of possible “false negatives” drop to 126. With the extended panel, the incidence of “false-negative” samples decreased to 7.6% while it was 10% with the base panel of antigens, corresponding to a reduction of false negatives of 23% (Figure 3).

Each of the four comparisons in Figure 3 are examples of *in vitro* HBT occurred between two individuals. In the first two cases (samples 23 vs. 27 and samples 5 vs. 35) we detected only one antigenic difference considering the base panel, thus giving an ATF result. However, by also considering the additional four antigens, the method detects two antigenic differences, thus leading to the possibility to match the criteria to report an AAF. In the two subsequent cases (samples 10 vs. 11 and samples 26 vs. 39), there are no antigenic differences detectable for the base panel, so the analysis of the samples would provide a negative result. By including in the analytical protocol also the additional antigens, at least one antigenic difference is found, which would make the samples suspect and would require a more in-depth analysis, suggesting the targeting/retesting of the athlete for a suspect transfusion procedure underway.

However, to our knowledge, a complete elimination of false-negative results in HBT detection seems to be achievable only by the implementation of alternative and very high specific methodologies, such as those based on DNA testing (Donati et al., 2013, 2021; Manokhina and Rupert, 2013; Stampella et al., 2016).

### Stability Study

Red blood cells have a lifespan of 120 days; thus, theoretically, they should be detectable in the recipient after transfusion for a maximum of 4 months. Once the transfusion has been performed, the ability to detect donor erythrocytes in the recipient's blood essentially depends on the amounts of erythrocytes transfused, on the quality of the blood sample, and on the time necessary to perform the analysis after the sample has been collected and delivered to the laboratory. In general, the storage at 4°C causes the blood sample to progressively loose its “analytical quality,” due to deterioration of the RBCs and haemolysis processes. We focused specifically on this last issue, and performed a stability study with the aim of monitoring the repeatability of the results of the flow cytometric analysis over time.

As shown in Table 2, results from the 11 mixtures prepared with the donor at 5% (and correctly detected as mixtures at *t* = 0 with 100% of the double population detected) showed no false-negative results after 2 month (although 2 samples were detected as “suspicious,” since only one antigen was recognized as composed of two erythrocyte populations). However, a reduction in the total number of double erythrocyte populations was detected compared to *t* = 0: at *t* = 1 (after 30 days of whole blood storage), 84% of the double populations were detected, and at *t* = 2 (after 60 days) 68% of the double populations were detected. More significantly, results from the five mixtures prepared with the donor at 0.5% (all of them correctly identified as mixtures at *t* = 0, with 100% of the double population detected), showed a negative result in two cases (mixtures #15 and #16) and an ATF in once case (#14) at *t* = 2. We observed a 44% and a 69% decrease in the detection of double populations, respectively, at *t* = 1 and *t* = 2. These data show that the relative percentage of the donor in the transfused sample has a significant impact on the stability of the results. A mixture with the donor at 0.5% simulates a situation in which an athlete is tested for HBT many days after the transfusion, when the level of the donor red blood cells has been progressively decreasing. Moreover, we observed that the loss of detectability of double populations affects DPN and DPE in comparable way (data not shown). Furthermore, our data also revealed that the ability to detect mixed erythrocytes populations over time depends on the intrinsic characteristics of the antigens examined: double populations on Big C, small c, Jka, and Jkb were still well-detectable even after 3 months, while double populations on Fya, Fyb, Big E, and S were not. This loss of detectability can be due to the following main causes: (1) RBC of the donor were no longer detected, since their concentration dropped below the limit of detection of the method, and (2) the two RBC populations of the donor and the recipient were no longer clearly detectable as separate clusters of events. We have determined that, for the mixtures at 0.5% (both DPN and DPE), the loss of detectability resulted solely as a loss of donor red blood cells. On the other hand, for the mixtures at 5%, the reason for the loss of information resulted more related to the quality of the double population: an insufficient separation of the peaks in the case of DPEs, also combined with an insufficient number of events in the case of DPNs.

**Table 2:** Result of the stability study over the 60 days period.

**Mixture**	**% Donor**	**big C**	**small c**	**big E**	**Jka**	**Jkb**	**Fya**	**Fyb**	**big S**		***t*** **= 0**	***t*** **= 1**	***t*** **= 2**
1	5	spe	DPN	spn	DPE	DPN	DPE	spe	spe		4	4	4
2	5	spe	DPN	DPN	DPN	DPE	DPN	spe	spe		5	5	3
3	5	spn	spe	DPE	DPN	DPE	DPE	DPN	spe		5	5	3
4	5	spe	DPN	DPN	spe	DPN	spe	spn	spn		3	2	1
5	5	DPE	DPN	DPN	spn	spe	spe	spn	DPN		4	4	3
6	5	spe	spe	spn	spe	spn	DPE	DPN	spe		2	2	2
7	5	DPN	spe	spn	DPN	DPE	DPN	DPE	spe		5	5	4
8	5	DPE	spe	spe	spe	DPN	DPN	DPE	spe		4	3	3
9	5	spe	DPN	DPN	spe	spn	DPE	spe	spe		3	1	1
10	5	DPE	spe	spn	DPE	DPN	DPE	DPN	DPN		6	4	4
11	5	spe	DPE	spn	spe	DPN	spe	DPN	spe		3	2	2
										Average	4.0	3.4	2.7
										Total	44 (100%)	37 (84%)	30 (68%)
12	0.5	spe	DPN	Spn	DPE	DPN	DPE	spe	spe		4	3	2
13	0.5	spe	DPN	DPN	DPN	DPE	DPN	spe	spe		5	5	2
14	0.5	spn	spe	DPE	DPN	DPE	spn	spn	spe		3	1	1
15	0.5	spe	spn	spn	spe	DPN	spe	spn	spn		1	0	0
16	0.5	spe	DPN	DPN	spn	spe	spe	spn	DPN		3	0	0
										Average	3.2	1.8	1
										Total	16 (100%)	9 (56%)	5 (31%)

*DPN, double population recipient non-expressing the antigen; DPE, double population recipient expressing the antigen; spe, single population expressing the antigen; spn, single population non-expressing the antigen. t1, after 30 days from t0; t2, 60 days after t0*.

### Improving the Detectability of Double Populations of Erythrocytes

**Figure 4:**
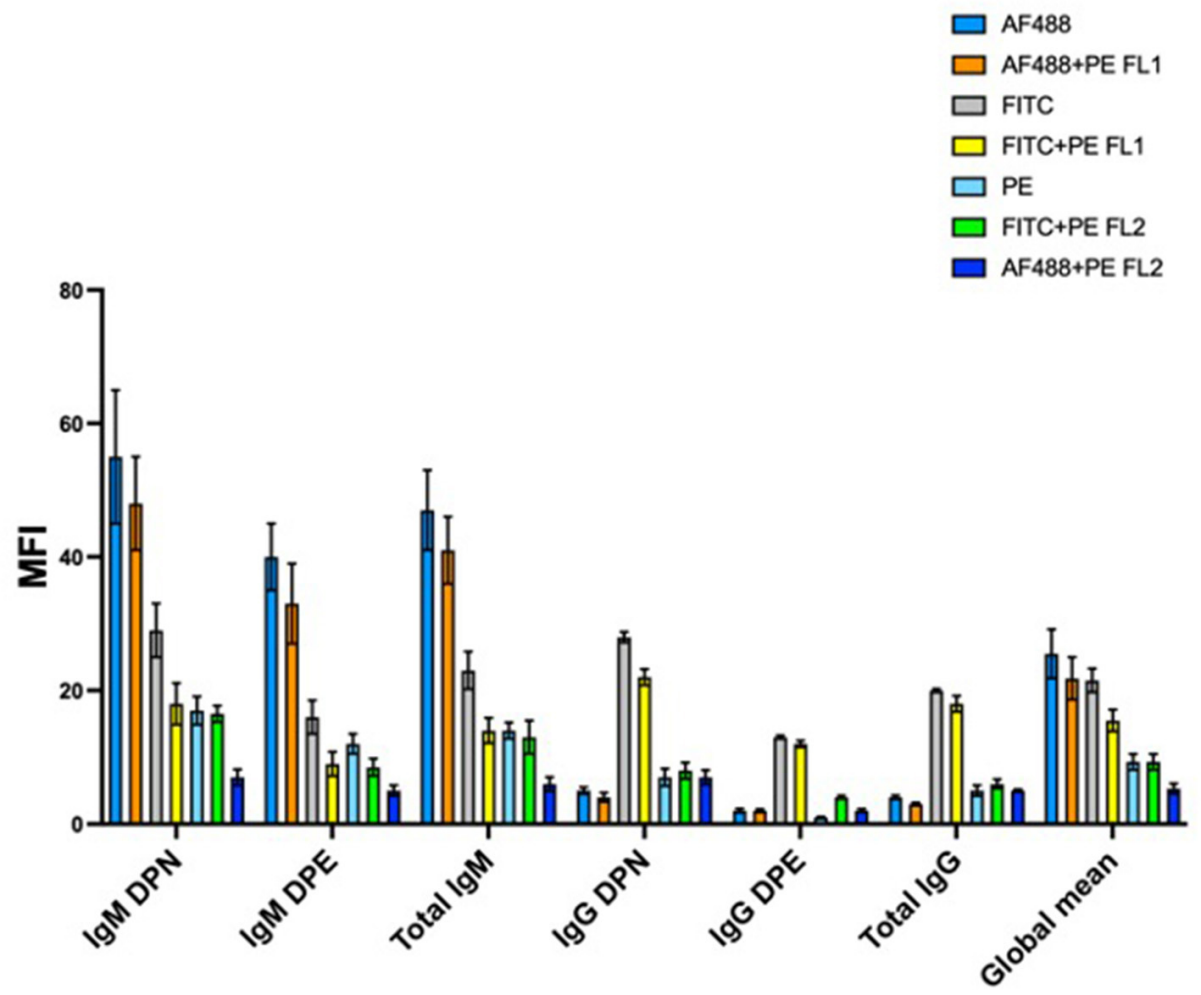
Mean separation of double population of erythrocytes for 10 mixtures prepared at 5% of the donor and stained with different fluorochromes and/or combination of fluorochromes. AF488, AlexaFluor 488; PE, phycoerythrin; FITC, Fluorescin isothiocyanate; FL1, flow cytometer detector 1 (522 nm); FL2, flow cytometer detector2 (585 nm); DPN, double population recipient non-expressing the antigen; DPE, double population recipient expressing the antigen. Subclass IgM antigens were big C, small c, big E, Jka, and Jkb, while subclass IgG antigens were Fya, Fyb, and big S.

One of the main requirements of the method is that the RBC population of the donor must be clearly separable and distinguishable from the RBC recipient population in the fluorescence histogram. This is not always easy to achieve, particularly when the number of antigenic sites of the donor and the recipient's RBC is not sufficiently different. Improvements in the detection of donor and recipient's RBC would allow to increase the general sensitivity of the method and, at the same time, to stem the occurrence of false negative results, which become more evident in the case a sample needs to be analyzed several days after the collection and after a storage period at 4°C, a quite common situation in the case of a B-sample analysis following an adverse analytical finding on the correspondent A sample. The application of a staining amplification technique has been demonstrated valid to improve the separation of cell populations of the donor and recipient (Voss et al., 2007). However, the staining amplification technique requires the use of additional antibodies and, in the end, it determines an increase in the number of staining incubation steps, which, in turn, leads to an increase in the overall analysis costs and times. Here we studied the benefit of the possible use of different staining fluorochromes conjugated on the secondary antibody, used individually or in combination. Figure 4 shows the result achieved on the global separation of double population of erythrocytes tested on 10 mixture samples prepared as 5% of the donor.

**Figure 5:**
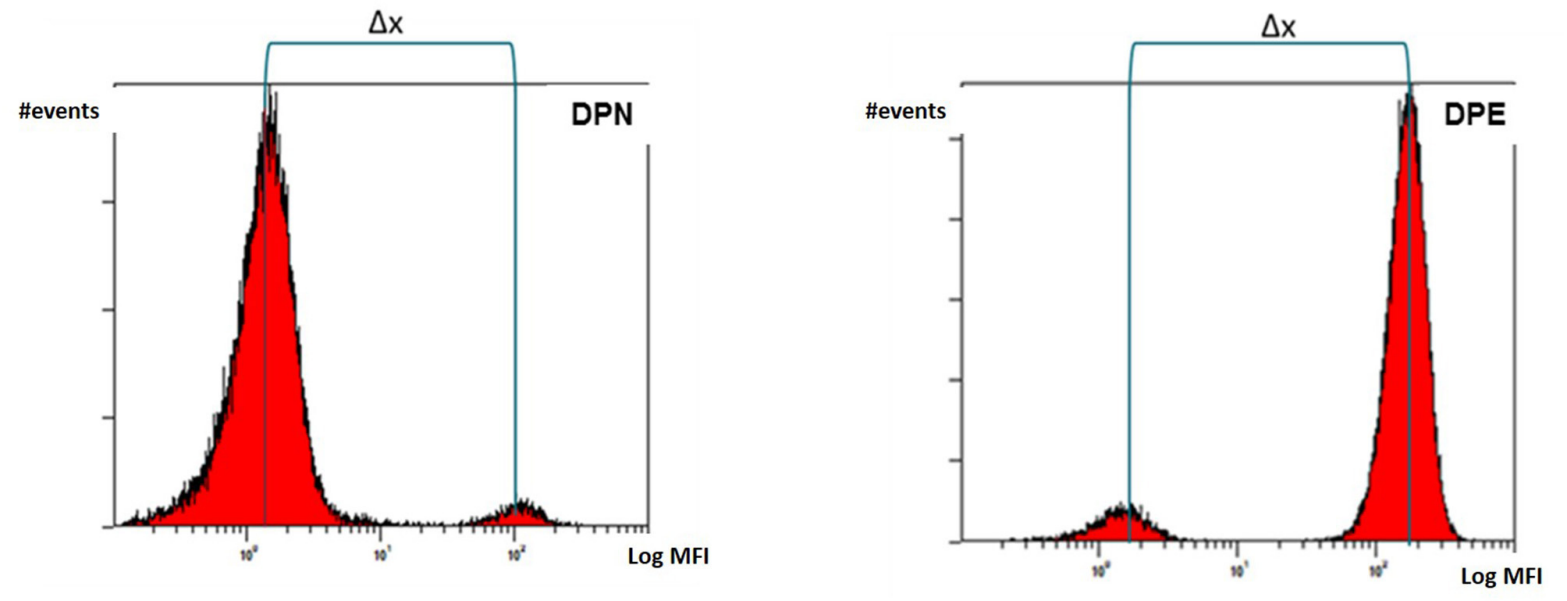
Separation of double population of erythrocytes (Δx) calculated as difference between median MFI values of the donor and recipient's peaks.

The separation of double population of erythrocytes (Δx) is calculated from the fluorescence histogram as the difference between the median MFI (Mean Fluorescence Intensity) of the peak with the higher fluorescence intensity (the donor in the DPN mixtures and the receiver in the DPE mixtures) and the peak with the lower intensity (the receiver in the DPN and the donor in the DPE) as illustrated in Figure 5.

We used secondary antibodies conjugated with Fluorescin Isothiocyanate (FITC), Phycoerytrin (PE), and AlexaFluor488 (AF488). FITC is typically the most commonly used fluorochrome in flow cytometry. It has a maximum of excitation at the wavelength of 488 nm and a maximum of emission at 525 nm. PE generally has a higher brilliance than FITC, it is always excited at 488 nm but it emits at a higher wavelength of 575 nm. AF488 represents a new generation of fluorochromes characterized by great brilliance and versatility. Their absorption wavelength is 495 nm and the emission occurs at 520 nm.

AF488 provided the best average separation when conjugated to a secondary antibody capable of binding IgM subclass antigens. This result was identical for both the DPN and DPE populations. The average separation obtained from the combination AF488 + PE (read on the FL1 detector which is centered on the wavelength of 525 nm) was high but not equal to the use of the AF488 alone. On the other hand, FITC fluorochrome, used alone or in combination with PE and read on FL1 detector, provided the best separation results for antigens of IgG subclass. A possible explanation can be given by the particular nature of the antibody subclasses. IgM antibodies are pentameric while IgG have only one antigen binding site. The superior brilliance of the AF488 results in better separation due to the additive effect due to the pentameric nature of the IgM. Overall, these results demonstrate how a flexible staining strategy in relation to the antibody subclass (IgM or IgG) and the type of double population to be detected (DPN or DPE) allows to improve the detection and consequently the analytical sensitivity. PE, despite being a very bright and strongly fluorescing fluorochrome, proved to return on average the worst separation between the double erythrocyte populations analyzed.

### Improved Gating Strategies

**Figure 6:**
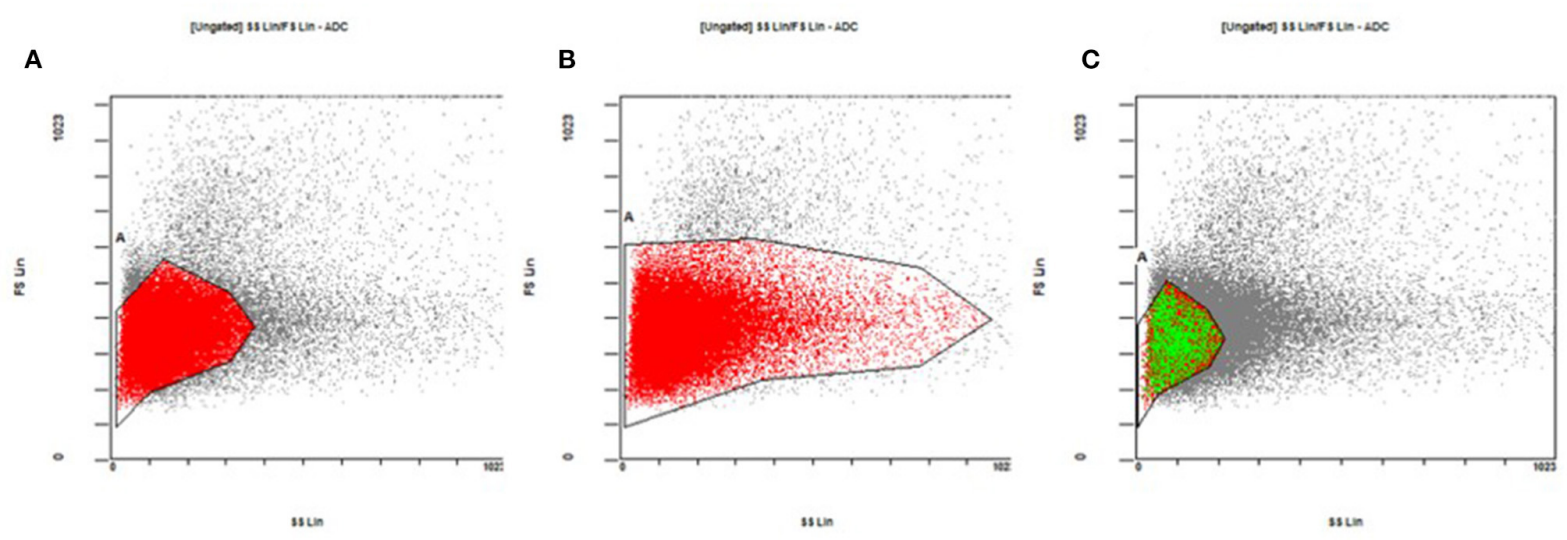
Gating strategies for the sampling of red blood cells on the size vs. complexity dual plot. **(A)** The core of the cloud of the events. **(B)** The core of the cloud and its entire tail, enclosing both mature and immature red blood cells. **(C)** The core of the red blood cell cloud, but limited to the area typical of the donor red blood cells (see the text for more details).

RBC gating areas are electronically set on the flow cytometer to circumscribe a sampling area on the size vs. complexity dual plot (Forwad Scatter vs. Side Scatter) so that to include only red blood cells and to exclude white blood cells, platelets and cell aggregates. We considered three different gating strategy (as described on Figure 6) applied on the analysis of 40 different RBC mixtures.

**Figure 7:**
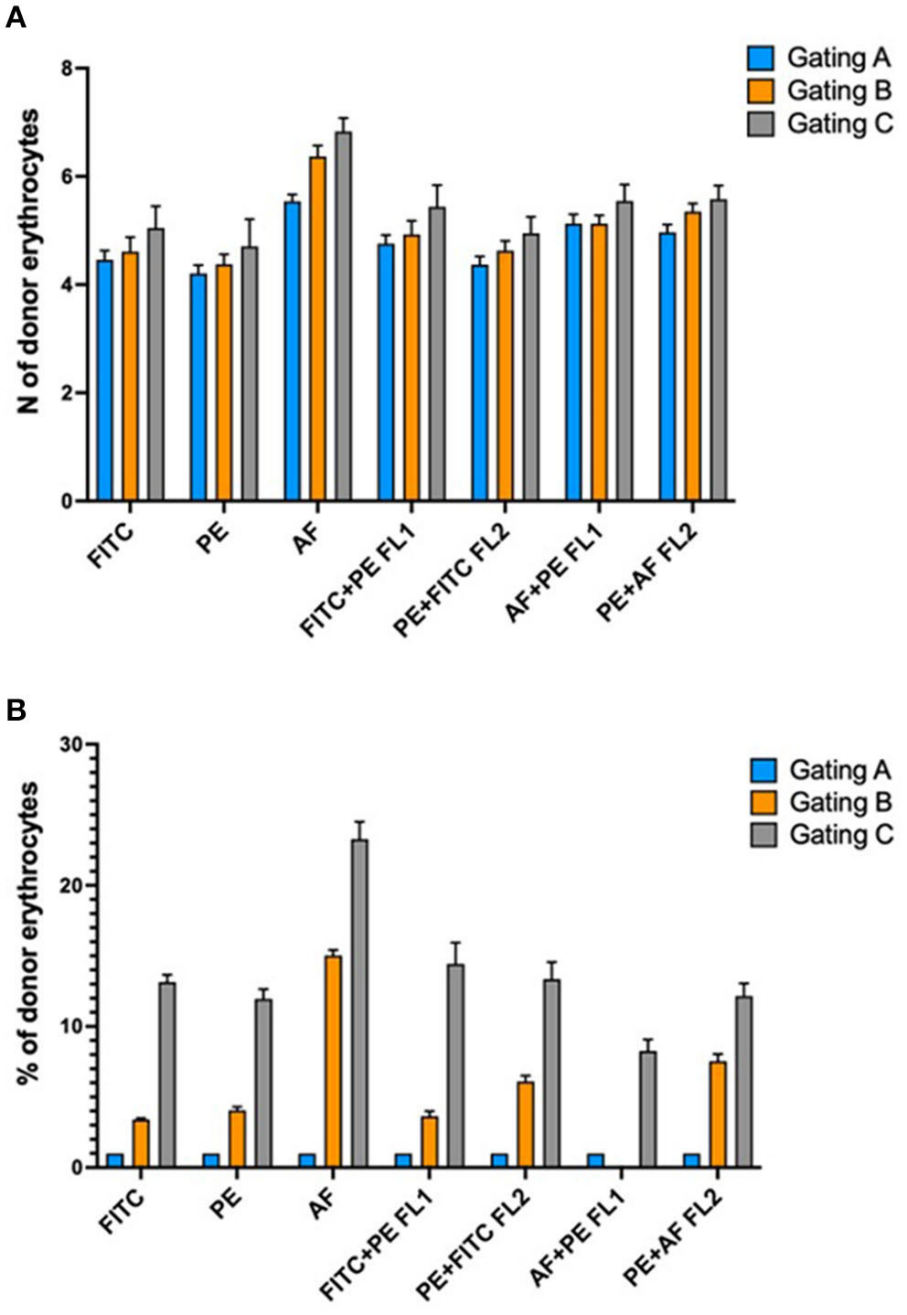
**(A)** Absolute increase in the number of detectable donor erythrocytes in relation to different gating strategies of the acquired events. **(B)** Percentage increase in the number of donor erythrocytes in relation to the different gating strategies. Explanation in the text.

Gating area A encloses the “core” of the cloud of the events on the plot where most of the mature red blood cells are found. Gating area B includes the core of the cloud and its entire tail and so enclosing both mature and immature red blood cells (reticulocytes). The gating area C includes the core of the red blood cell cloud but this time reduced only in the area where the donor red blood cells typically are found. By narrowing the sampling area around the donor cells (gating C) we obtained an increase in the number of donor cells detected and consequently a percentage increased on the total number of 50,000 events sampled in the area (Figure 7A). This result is consistent for each fluorochrome used even though the greatest relative increase occurred with the use of the AF488 fluorochrome (Figure 7B). This “tailored” gating strategy has therefore proved effective in improving number of detectable donor cells which is one of the positivity criteria required by the method.

## Conclusions

Flow cytofluorimetry remains a versatile, practical and sensitive technique for the detection of the abuse of HBT in the sport doping. This study addressed some of the limitations of the method, aiming to reduce the overall risk of false-negative samples. The results may be summarized as follows:

1) Differences in the expression frequencies of some antigens is due to the belonging of individuals to different ethnic groups. It follows that the most suitable panel of antigens to be used for the screening must to be created considering the antigen diversity on a regional scale. As we have seen, the antigens of the Duffy group (Fya and Fyb) are perfectly suitable for the screening of blood samples of European/Caucasian athletes but of little use for the screening of samples of athletes of east Asian origin. However, it must be considered that for large international events (i.e., such as Olympics), a panel comprising the highest number of antigens must necessarily be considered due to the participation to the event of athletes of all ethnicities.
2) By increasing the number of antigens to be screened, the result is a safe and ascertained reduction in the number of “false-negative” samples and thus an improvement of the sensitivity of the method. In this study, by expanding the antigen screening panel from 8 to 12, we obtained a 23% reduction of “false-negatives.” The four supplementary antigens we selected for the panel extension, have proven to be adequate for the discrimination of mixed RBC populations, allowing a staining comparable to that of the antigens already in use in the base panel. However, it must be also considered that a further expansion of the antigenic panel (by considering antigens such as M, N, P1, and Lewis a/b) might be excessively costly in terms of reagents and analysis times.
3) The storage of samples at 4°C causes a reduced capability of detection of double erythrocyte populations, which is more critical for the mixtures containing a small percentage of the donor's red blood cells. Although the deterioration of the blood sample can have different dynamics depending on the single individuals, in the case of an adverse analytical finding it would be advisable to carry out the B-analysis in a reasonably short time (however not later than 1 month after the collection of the blood sample) in order to preserve the reliability of the results.
4) The specificity and the sensitivity of the method can be improved by applying targeted staining strategies depending on the type of double erythrocyte population to be detected. A tailored RBC gating area allows to increase the detection of the absolute number and the relative percentage of donor transfused cells in the recipient sample. This becomes essential in all those samples subjected to the screening test at a distance of time from the transfusion occurred and in which the most part of the donor RBC were disposed by the reticuloendothelial system of the recipient. Moreover, the use of secondary antibodies with different fluorochromes in relation to the different subclass of the antigen to be detected, improves the separation of the donor and recipient cells. This circumstance becomes decisive in all those cases in which the red blood cells of the donor and the recipient differ in a very small number of antigenic sites. In any case, the intra-laboratory validation of the method is the essential aspect to produce accurate and reliable results over time. Laboratories must take into consideration the instrumental background levels, the specificity and sensitivity performance of each antigen tested, the best erythrocyte gating strategy, and the absence of possible carryover between the analyzed samples.

## Data Availability Statement

The raw data supporting the conclusions of this article will be made available by the authors, without undue reservation.

## Author Contributions

FD: conceptualization, experimental planning and supervision, data processing and reviewing, writing, original draft, and review and editing. XT: experimental planning and supervision, data reviewing, and writing—review and editing. SP: analytical investigation, data collection, pre-evaluation, stability studies, writing, and original draft. DP: conceptualization, experimental planning and setup, analytical investigations, data reviewing, writing, original draft, and review and editing. GP: analytical investigation, data collection, pre-evaluation, antibody comparison studies, writing, and original draft. FB: conceptualization, supervision, project administration, resources, writing—review and editing, and submission. All authors contributed to the article and approved the submitted version.

## Funding

This study was funded by the Laboratory Internal Research Funds.

## Conflict of Interest

The authors declare that the research was conducted in the absence of any commercial or financial relationships that could be construed as a potential conflict of interest.

## Publisher's Note

All claims expressed in this article are solely those of the authors and do not necessarily represent those of their affiliated organizations, or those of the publisher, the editors and the reviewers. Any product that may be evaluated in this article, or claim that may be made by its manufacturer, is not guaranteed or endorsed by the publisher.
